# Validating attentive locomotion training using interactive treadmill: an fNIRS study

**DOI:** 10.1186/s12984-018-0472-x

**Published:** 2018-12-20

**Authors:** Seunghue Oh, Minsu Song, Jonghyun Kim

**Affiliations:** Department of Robotics Engineering, DGIST (Daegu Gyeongbuk Institute of Science and Technology), 333 Techno Jungang-daero, Daegu, 42988 Republic of Korea

**Keywords:** Gait training, Attention, Task complexity, Walking speed, Interactive treadmill (ITM), fNIRS

## Abstract

**Background:**

Existing treadmill-based locomotion training, which has been used for gait function recovery, still has limitations, such as less attentive training. Interactive treadmills (ITMs) were developed to overcome these limitations, but it has not yet been verified that ITMs can make the user pay closer attention to walk training.

**Methods:**

An experimental comparison between ITMs and conventional treadmills was conducted by measuring the level of the user’s attention using functional near-infrared spectroscopy (fNIRS). To consider the effect of task complexity on the subject’s attention, we provided two (slow and fast) speed conditions for walking on both treadmills.

**Results:**

Both the cortical activity images and oxygenated hemoglobin (oxyHb) changes showed that the level of attention to walking induced by the ITM was significantly higher than that induced by the conventional treadmill. We found that the walking speed on the ITM also affected the level of attention.

**Conclusion:**

ITM-based locomotion training would be a promising solution to the limitations of existing treadmill-based locomotion training currently used to improve gait function recovery.

**Trial registration:**

DGIST-HR-150309-03-02. Registered 01 March 2015.

## Background

Recovery of gait function is a typical goal of rehabilitation [1, 2], and thus gait training for patients with impaired gait has been widely used in clinical practice [2–5]. Although the ultimate goal of gait training is to improve overground walking ability [6], gait training has mainly been conducted on treadmills [5, 7]. This is because treadmills can safely and consistently provide task specific training in a limited space [8, 9], and treadmill walking is not significantly different from overground walking with respect to kinematic and kinetic aspects [10, 11]. However, treadmill-based locomotion training (TBLT) still has limitations on both physical and mental activity.

### Limitations on physical activity

The kinematic and kinetic similarities between treadmill walking and overground walking were only verified with the treadmill belt turning at a constant speed [10, 11]. Since existing TBLT is mainly focused on walking at a constant speed [1, 5], walking on the treadmill can be regarded as similar to walking overground at a constant speed. However, it is clear that treadmill walking is a different physical activity compared with actual overground walking in which users decide their walking speed by themselves [8].

### Limitations on mental activity

It is well-known that walking with a normal gait is a highly automated skill developed by natural practices during the growth period [12, 13]. Hence, except for special circumstances (i.e. walking on ice), a healthy person can walk well using very little attention on walking [13, 14]. However, for patients with impaired gait due to central nervous system injuries, it is difficult to maintain their normal gait, and thus it is necessary to re-establish their gait for locomotion [13]. Previous studies reported that a patient’s mental activity during gait re-establishment, especially the external focusing of attention [15, 16], such as focusing on regulating gait speed, resulted in a better outcome for training [14–16]. In spite of the importance of the patient’s attention being properly focused during rehabilitation, existing TBLT has been vulnerable to a lack of properly focused attention. TBLT, with a constant treadmill speed, provides a monotonous task for the user, who can easily become accustomed to the task, and would therefore be less attentive [8, 9].

There have been some attempts to overcome these limitations of existing TBLT. Several self-paced treadmill systems (i.e. TreadPort [17] and treadmill-on-demand [18]) were reported to enable the user to adjust the treadmill walking speed by using a treadmill belt speed controller based on the user’s position measured either by an active mechanical tether [17, 19] or a non-contact sensor [18], or force sensors [20]. They commonly allow the user to accelerate or decelerate on the treadmill, and thus treadmill walking becomes more similar to overground walking in terms of physical activity. Along with the self-paced treadmill function for physical activity, they also provided external visual biofeedback, which would be beneficial to overcome the limitations of TBLT on mental activity [17, 19, 20]. However, these studies did not focus on the mental activity, so did not verify whether the attention, which is related to the mental activity, was really improved or not.

Recently, our research team has been developing an interactive treadmill (ITM) [8, 9, 21] as an effective and efficient system when used with TBLT for gait rehabilitation. To overcome the limitation on physical activity cost-effectively, the self-paced treadmill function of the ITM was implemented based on a novel belt speed controller for minimizing the anomalous force generated by the user’s acceleration or deceleration [8]. Moreover, thanks to the self-paced treadmill function, the ITM has provided an environment that causes the user to be more attentive during treadmill walking. Based on the assumption that proper biofeedback during locomotion training would improve the user’s attention, this is achieved by including a protocol that causes the user to make efforts to achieve a target walking speed, which can be regarded as giving the user an external focus of attention [16], through visual biofeedback. Although providing an attentive environment is the main goal of the ITM, the effect of the ITM regarding the user’s attention has not been verified yet, as the existing self-paced treadmill system did not [17, 19, 20]. It should be noted that there was an attempt to check the brain activation during treadmill walking on ITMs, but it did not investigate whether the attention was improved or not [22].

Based on the hypothesis that ITM-based locomotion training increases the user’s attention to the training, the purpose of this study is to investigate whether this hypothesis can be confirmed or not. For that, we used functional near-infrared spectroscopy (fNIRS), a non-invasive method that indirectly monitors brain activation by measuring changes of hemoglobin (Hb) in cerebral blood flow [23, 24], to observe what brain activation occurs in the cortical area related to gait. Compared with similar modalities to measure Hb changes, i.e. functional magnetic resonance imaging and positron emission tomography, the main advantages of fNIRS are 1) more ecological setting without strong immobility constraints [25, 26] and 2) less susceptible monitoring to motion artifacts as well as metallic materials [27]. Due to those advantages, fNIRS has been regarded as a promising neuroimaging technique for complex behavior with active motion [28–31], especially for locomotion/walking [25, 26, 32]. Hence, fNIRS has been widely used to observe brain activation in walking [25, 26, 32–38]. Moreover, fNIRS was widely applied to specific tasks regarding mental activity, such as cognitive-related tasks [31, 39–41] and dual task execution [31, 36, 37]. Based on the reports that the degree of mental activity, such as the level of attention due to task complexity, is closely related to the change of oxygenated Hb (oxyHb) [29, 39], fNIRS can also be used to measure the level of attention [36, 37, 41, 42]. Therefore, we have used fNIRS to investigate the user’s attention to walking during ITM-based locomotion training, by measuring the change of oxyHb in several brain regions which are related to this type of attention.

## Methods

### Interactive treadmill

**Fig. 1:**
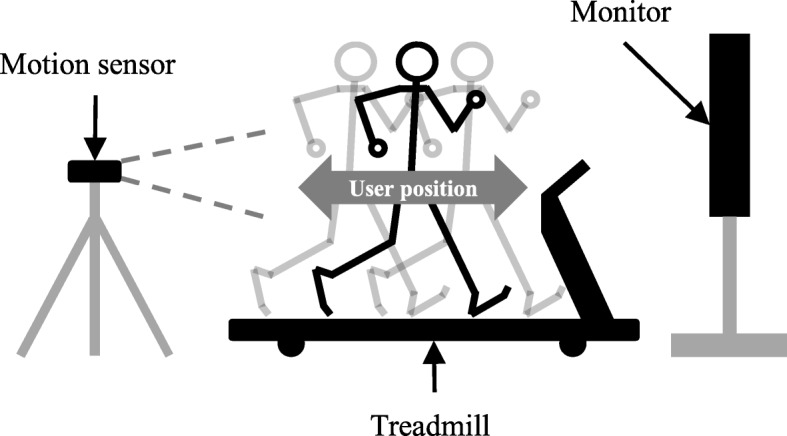
Schematic diagram of interactive treadmill system

The ITM consists of a motion sensor (Kinect v1, Microsoft, USA), a treadmill (PPS MED, Woodway, USA), a treadmill belt speed controller, and a monitor to provide biofeedback (Fig. 1) [8, 9, 21]. In order to overcome the limitation of existing TBLT in terms of physical activity, the ITM enables the user to stay on the belt even when undergoing acceleration or deceleration. This is accomplished through the belt speed controller which uses feedback from the user’s position, which is measured by the motion sensor. Moreover, the controller of the ITM, which can minimize the anomalous forces generated by the belt acceleration, allows the user to perform a more natural acceleration or deceleration, similar to overground walking [8].

**Fig. 2:**
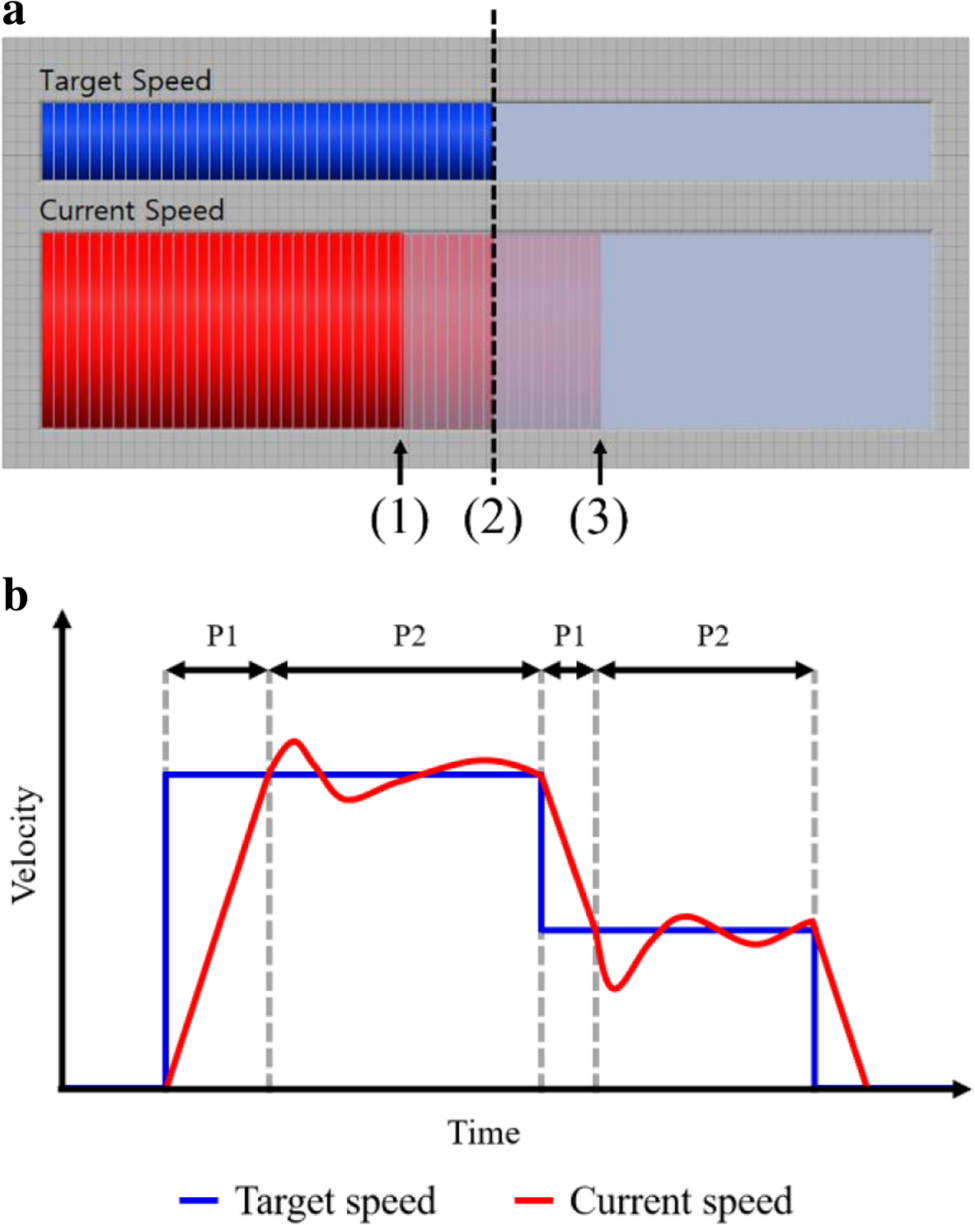
**a** Visual feedback of ITM. (1) User needs to accelerate. (2) User needs to maintain the speed. (3) User needs to decelerate. **b** Example of ITM locomotion training profile. P1 is period #1. P2 is period #2

In addition, based on the user’s natural acceleration or deceleration on the ITM, we proposed the following gait training protocol to overcome the limitation of TBLT in terms of mental activity. The user can recognize his or her current walking speed and the target speed in real time by using the visual biofeedback displayed on the monitor (Fig. 2a), and they can attempt to match their speed to the target speed during the walking task. If the target speed is provided as shown in Fig. 2b, the user needs to do the following to achieve the target speed: 1) reach the speed through rapid acceleration or deceleration (period #1 in Fig. 2b), and 2) maintain the speed with gait adjustment (period #2 in Fig. 2b). Note that the ITM requires the user’s effort to maintain a constant walking speed. After all, the ITM was proposed as a simple and promising solution to overcome the limitations on mental activity during TBLT, because it provides a protocol to induce the user’s continuous attention during TBLT, based on the characteristics of the ITM [8, 9, 21].

### Experimental setup

In this study, we compared the ITM with the conventional treadmill (CTM) to evaluate the performance of the ITM on inducing the user’s attention. It is reported that the level of the attention is closely related to the task’s complexity [39], and walking speed is a key factor of the task complexity when walking. Hence, we attempted to compare the levels of attention when using the ITM and the CTM with same walking speed. For that, with two target speeds (slow and fast), the comparison consisted of four tasks: ITM-slow, ITM-fast, CTM-slow, and CTM-fast. Note that the target speeds of the CTM were the speed of the treadmill belt provided.

**Fig. 3:**
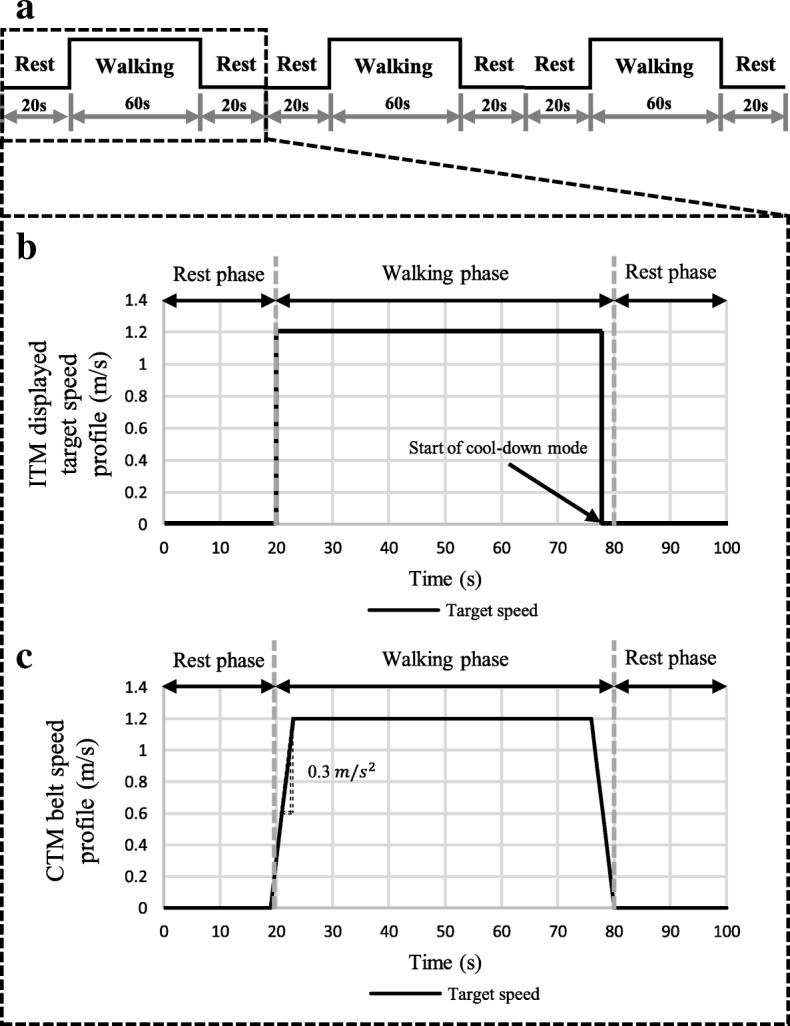
**a** Block paradigm design. **b** Example of ITM displayed target speed profile. **c** Example of CTM belt speed profile

For each task, a block paradigm design that repeats three cycles, (with each cycle consisting of three phases, rest (20 s)-walking (60 s)-rest (20 s)), was used to measure brain activation, as illustrated in Fig. 3. During the ITM-slow and ITM-fast tasks, we provided a target waking speed during the walking phase. During the cool-down mode, the ITM was activated to stop the ITM belt before the end of walking phase and the ITM displayed a zero-target speed (Fig. 3b). On the other hand, the belt speed profile which is to mimic the walking behavior of the ITM, as shown in Fig. 3c, was implemented for CTM-slow and CTM-fast tasks. The magnitude of the acceleration and deceleration of the profile was set to 0.3 m/s^2^ (Fig. 3c).

The reason we conducted the experimental comparison with two different walking speeds (slow and fast) was to verify how the task complexity felt by the user affects the user’s level of attention induced by the ITM. For that, before the experiment, we obtained each subject’s preferred walking speed by calculating his or her average speed during a 10 m overground walk, and the slow and the fast walking speeds for each subject were determined as follows: the fast speed was set to 125% of the preferred speed, and the slow speed was 75% of the preferred speed [8]. The difference in the user’s level of attention when using the ITM or the CTM needed to be compared under similar work load intensity by considering the individual’s gait capacity [43]. The preferred speeds are different for each subject, and thus the task complexity felt by the subject would not be same if fixed target speeds were provided to all subjects.

### Participants

**Fig. 4:**
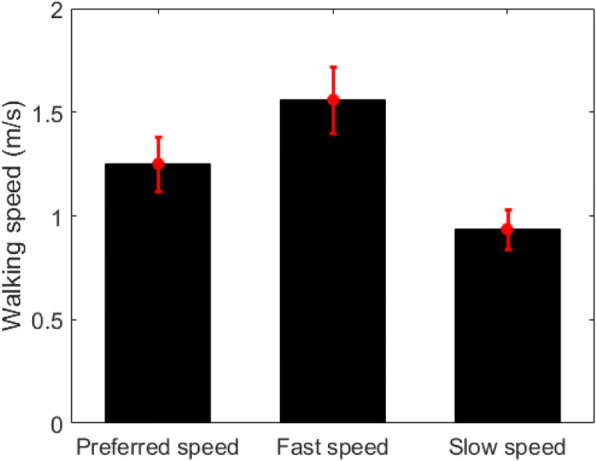
Preferred walking speeds measured on a 10 m overground walk

Twenty healthy subjects, who did not have any musculoskeletal or nervous system disease, were recruited. The participant demographics were summarized in Table 1. All participants were given the full instructions of the study and agreed to participate in the experiment. The experiment was conducted with the IRB (DGIST-HR-150309-03-02) approval of DGIST (Daegu Gyeongbuk Institute of Science and Technology). Figure 4 shows the subjects’ preferred walking speeds as measured by a 10 m overground walk.

**Table 1:** Participant demographics

Characteristics		
Age (years)	27.4 ± 2.54; range: 21–34	
Gender	male: 16	female: 4
Physical activity level	healthy (no underlying orthopedic or neurological disorders)	
Preferred speed (m/s)	1.25 ± 0.13	
Education level (years)	17.5 ± 1.9

### Apparatus

**Fig. 5:**
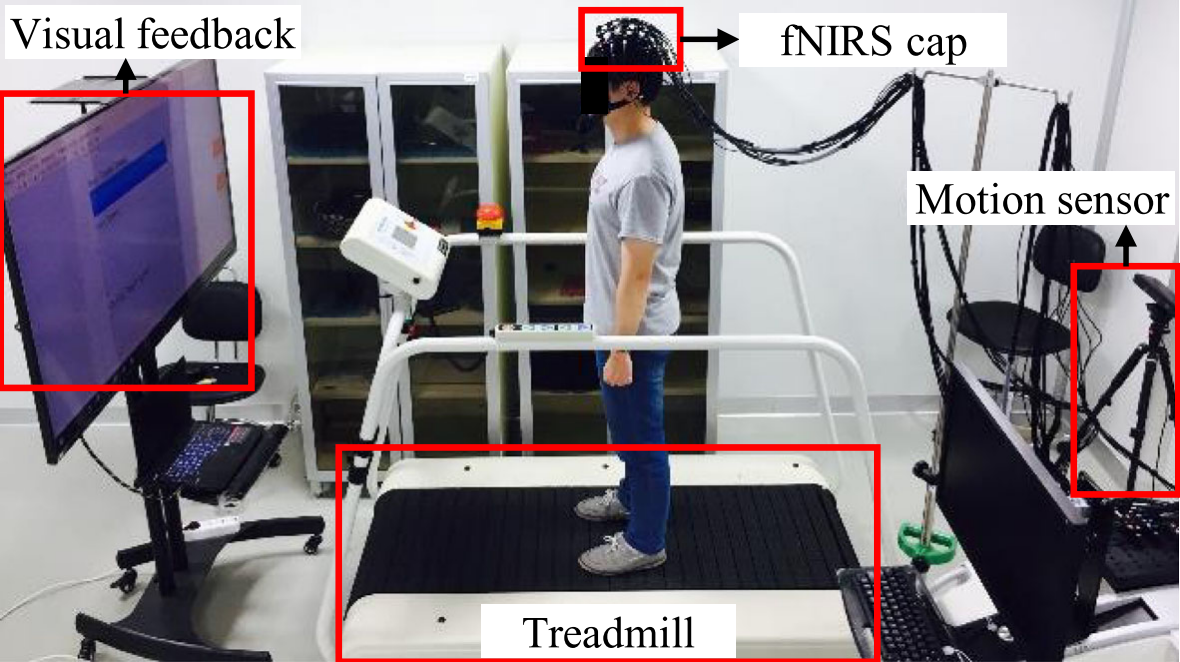
Experimental setup

The ITM system used for this experiment is shown as Fig. 5. For the comparison between the ITM and the CTM, the treadmill of ITM system (without self-paced treadmill function) also used as CTM. Figure 3c shows the belt speed profile of CTM.

By using a fNIRS system (LABNIRS, Shimadzu, Japan) that has continuous-wave laser diodes with 780, 805 and 830 nm wavelength, the subject’s cortical activation was observed at a sampling rate of 16 Hz [44]. We used a well-known international 10–20 system to locate the fNIRS optodes [45]. The cranial vertex (Cz) was set as a reference point for the optode placement. For accurate fixation, the optodes were mounted on the holder in the NIRS cap, and the distance between the pair of light source and detector optodes was set to 3 cm. After acquisition of the fNIRS data, the coordinates of all the optodes and the positions of the anatomical landmarks (nasion, Cz, left and right pre-auricular points) were obtained by using Fastrak System (TX-2, Polhemus, Colchester, VT, USA), based on the Montreal neurological institute standard brain space [46].

**Fig. 6:**
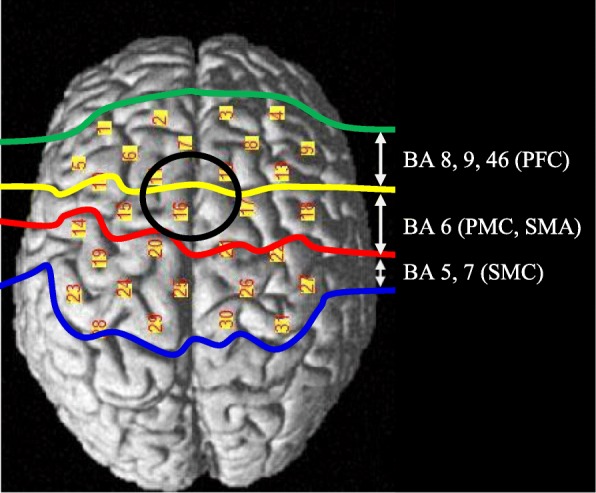
NIRS channels and regions of interest (ROI)

The purpose of this experiment is to observe the subject’s level of attention to walking. It is well-known that the cognitive region of the prefrontal cortex (PFC) is related to general attention [36, 41, 47]. However, we cannot guarantee that by observing the PFC the attention measured is always the attention to walking because the subject could perform dual tasks while walking by mistake. Thus, the additional features, which are related to the attention, need to be monitored; task complexity felt by subject would determine the level of attention [39], and variability of gait parameters could possibility cause the subject to focus the attention which is measured [48]. Therefore, we chose the region of interest (ROI) based on the Brodmann area, as follows: cognitive region of the prefrontal cortex (PFC) [36, 41, 47], sensorimotor cortex (SMC) (which is related to task complexity) [39, 42], and supplementary area (SMA) and premotor cortex (PMC), which are related with variability of gait parameters [43, 48]. In order to measure those regions, 20 nodes (5 × 4) with 31 channels were used, as illustrated in Fig. 6.

### Protocol

After determining the subjects’ target speeds based on his or her preferred walking speed measured prior to the experiment, we attached the fNIRS optodes to the subject who was sitting comfortably in a chair and wearing a NIRS cap on his or her head. After that, the subjects were asked to try about three minutes of walking on the ITM and the CTM to become accustomed to those experimental environments. The subjects conducted four tasks (ITM-slow, ITM-fast, CTM-slow, and CTM-fast) in randomized order. Each task was performed with the block paradigm design (Fig. 3a), as follows. When each task started, the subject stood on the treadmill for 20 s as a rest phase (rest in Fig. 3a), then walked for 60 s after we gave the verbal instruction, ‘start’ (walking in Fig. 3a). During the walk on the ITM, the subjects were instructed to match their walking speed to the target speed by using the visual biofeedback provided by the monitor which displayed the walking speed as well as the target speed in real-time (Fig. 2a). As to the walk on the CTM, the treadmill belt moved at the pre-defined speed profile (Fig. 3c) and there was no display on the monitor. Note that the subjects were allowed to shake their arms naturally during walking. After that, the subject waited for 20 s while standing on the treadmill until the next walking phase started (rest in Fig. 3a). That rest-walking-rest cycle was repeated three times for each task (Fig. 3a).

### Data analysis

We have mainly used oxyHb for data analysis because oxyHb is known to be sensitive to task-related changes [33, 49]. Based on the oxyHb measured by fNIRS, we obtained optical imaging of cortical activities to show the location of the activation, and calculated the changes of oxyHb which can represent the degree of cortical activation quantitatively. NIRS_SPM, a MATLAB-based software package for statistical analysis of fNIRS, was used [46], and Gaussian smoothing with a full width at half maximum of 2 s was applied for noise compensation. In addition, Wavelet-MDL-based detrending was used to compensate for motion artifacts due to the subject’s movement, such as breathing [50]. General linear model (GLM) analysis with a canonical hemodynamic response curve was performed to model the hypothesized HbO response under the experimental condition [46, 50].

For group analysis of the optical imaging of cortical activities, the interpolated activity map over the cortical surface from the T statistics calculated at discrete channels were computed by using NIRS-SPM, and for a stricter analysis, HbO was considered significant at *p* < 0.05. The oxyHb changes for the regions of interest (PFC, SMC, SMA, and PMC), which were expressed in millimolar-millimeter units, and were obtained by summing the oxyHb changes of the channels included in the respective region. Here, the oxyHb change of each channel was calculated by subtracting the average oxyHb in the initial 10 s range of the rest interval from the average oxyHb in all task intervals. By using SPSS software (SPSS Inc. released 2006. SPSS for Windows, Version 15.0, SPSS Inc., Chicago, IL, USA), one-way ANOVA was applied to determine whether there was a statistically significant difference in the oxyHb change in each region of interest due to task differences (*p* < 0.05). In addition, we calculated the effective size(*d*) to evaluate the difference [51]. In general, the effect size (*d*) is generally not important if *d* < 0.2, small ≥0.2, medium ≥0.5, large ≥0.8 and very important when it exceeds 1 [41].

## Results

### Optical imaging of cortical activities

**Fig. 7:**
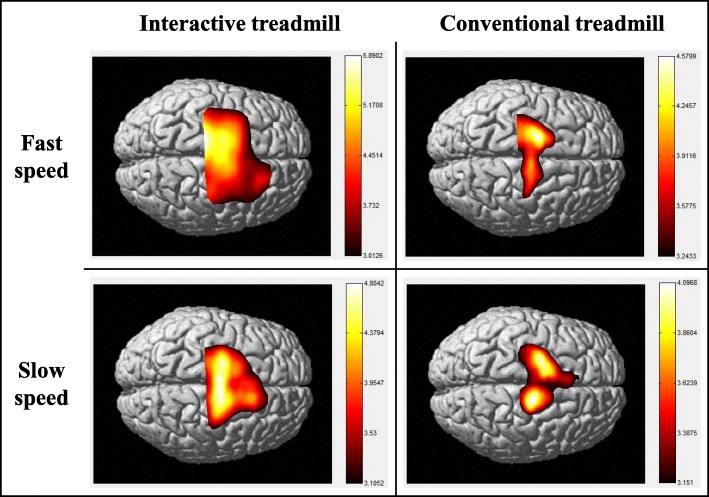
Optical imaging of cortical activities (group analysis)

The group analysis result of the optical imaging of cortical activities is shown in Fig. 7. Except for the CTM with fast speed, significant cortical activation was observed in all ROIs. Note that there was cortical activation of the SMC, SMA, and PMC regions when measuring the CTM with fast speed (Fig. 7). Regardless of walking (target) speed, cortical activation under the ITM occurred in a broader area than that in the CTM (Fig. 7). As to the PFC region related to the attention [36, 41, 47], the ITM always showed the activation while the CTM did not with fast speed, and with slow speed, the activation under the ITM covered a broader area of the PFC region than it did in CTM.

### Change of OxyHb

**Fig. 8:**
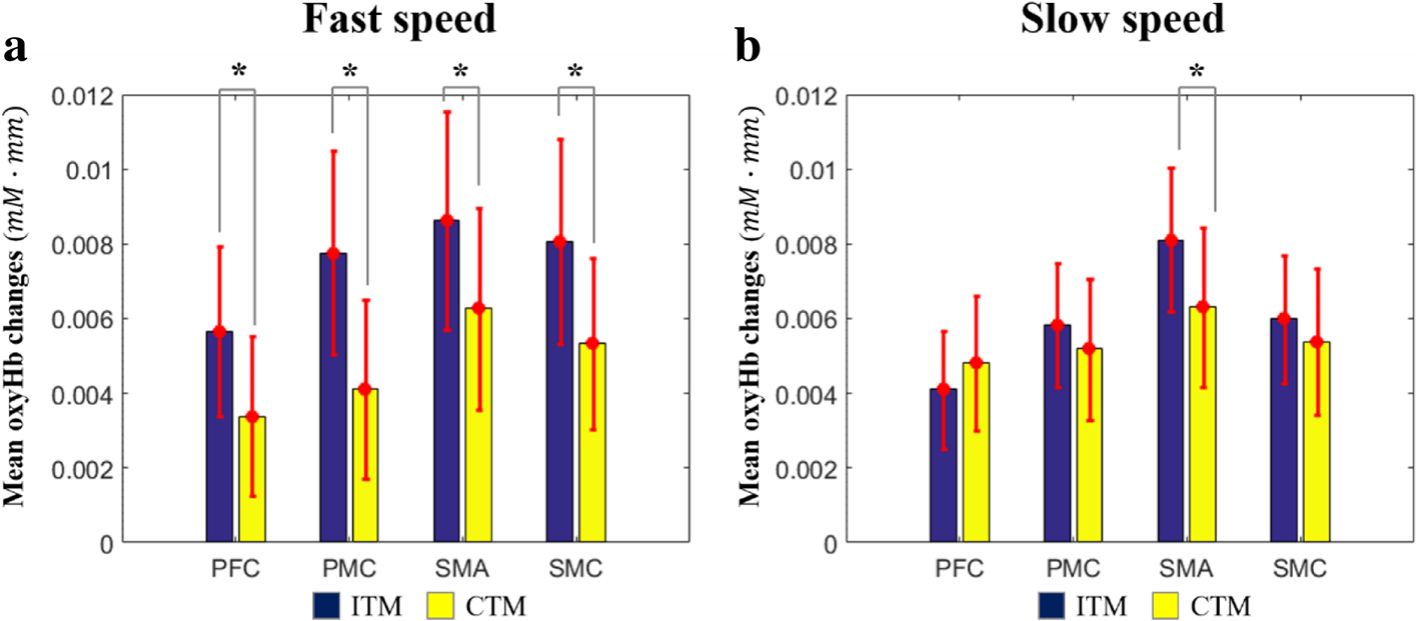
Mean oxyHb changes. PFC: Prefrontal cortex, PMC: Premotor cortex, SMA: Supplementary motor cortex, SMC: Sensory motor cortex, oxyHb: oxy-hemoglobin, *mM · mm*: millimolar – millimeter

Figure 8 shows the comparison of OxyHb changes between the ITM and the CTM. First, in the fast speed, the oxyHb change in the ITM was larger than that in the CTM, and the differences of the oxyHb changes between the ITM and the CTM were significant in all regions (*p* < 0.05) (PFC: F = 2.70, *d*=1.38; SMC: F = 2.45, *d*=1.37; SMA: F = 2.28, *d*=1.08; PMC: F = 3.09, *d*=1.84) (Fig. 8a). In the slow speed, the oxyHb change of the ITM was larger than that of the CTM in the PMC, SMA, and SMC regions, but the differences were not significant except in the SMA region (*p* < 0.05; F = 2.21, *d*=1.24) (Fig. 8b).

**Fig. 9:**
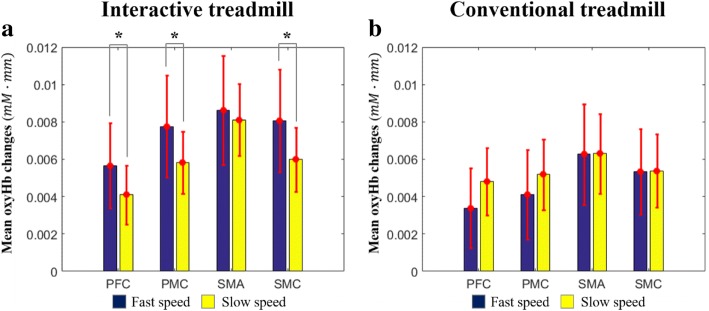
Mean oxyHb changes. PFC: Prefrontal cortex, PMC: Premotor cortex, SMA: Supplementary motor cortex, SMC: Sensory motor cortex, oxyHb: oxy-hemoglobin, *mM · mm*: millimolar – millimeter

To investigate the effect of walking speed, we checked the oxyHb change in each treadmill environment according to target speeds (slow and fast speeds). The ITM showed larger oxyHb increases in fast speed than in slow speed, and the difference of the oxyHb increases was significant in the PFC, SMC and PMC regions (*p* < 0.05) (PFC: F = 2.19, *d*=0.94; SMC: F = 2.27, *d*=1.04; PMC: F = 2.27, *d*=0.98) (Fig. 9a). On the other hand, the CTM did not show any significant difference in all regions (Fig. 9b).

## Discussion

From the experimental comparison, we found that both the optical imaging of cortical activities and change of oxyHb are supporting our hypothesis; ITM walking increases the user’s attention to walk, compared with CTM walking.

In the optical imaging of cortical activities shown in Fig. 7, one can see that the broader activation of all ROIs (PFC, SMC, SMA, and PMC) occurred under the ITM. Since the PFC and SMC regions are related to attention (control of attentional resources) [36, 41, 42, 47] and task complexity with voluntary movement [39], respectively, this broader activation of the PFC and SMC regions represents the subject’s increased attention under the ITM walking. Of course, the increased attention measured could be different from attention to walking. However, the broader activation of the SMA and PMC regions, which are related to variability of gait parameters [48], can be regarded as indirect evidence that walking is the focus of the increased attention because the activation of the SMA and PMC regions would come from the effort to match the target speed by modulating the gait parameters. It is noteworthy that there was no PFC activation under the CTM with fast speed. This lack of activation under the CTM represents the current characteristics of existing TBLT.

The results of the oxyHb changes also show that the ITM causes more attention to be used for walking than the CTM. The significant oxyHb increase of all ROIs under the fast speed clearly shows a larger level of attention under the ITM than that under the CTM (Fig. 8a). On the other hand, under slow speed, the significant increase occurred in the SMA region only (Fig. 8b). From the functions of the ROIs (i.e. SMC) [39], it would be because the subjects feel there was no significant difference of task complexity between the ITM and the CTM walking due to slow speed, while the ITM still needs more effort to maintain the target speed by modulating gait parameters than the CTM. This result implies that trying to make the user give more attention to walking (training) with the ITM-based locomotion training would be more effective with a fast target walking speed. It should be noted that the fast target speed is not absolute but relative, according to the user’s preferred speed (or gait capacity).

The oxyHb increases of most ROIs under the fast target speeds (Fig. 9a) also supports the characteristics of the ITM above, the relationship between the attention to walking and the target walking speed. In contrast, the CTM did not show any difference according to walking speeds (Fig. 9b), which was already reported [35]. This result shows that the ITM can induce attentive training by providing appropriate target walking speed, which cannot be done with the CTM.

The dual task with walking could be more attentive than simple walking. There were several fNIRS studies to investigate the effect of the dual task, and their results showed larger brain activities than simple walking [32, 37, 52, 53]. Moreover, similar result was reported in a study with a different imaging modality, transcranial doppler (TCD) [54]. Note that TCD was applied to a cognitive-related task or dual task in walking in order to monitor brain activation by measuring cerebral blood flow [54, 55]. Those results are consistent with our result that ITM walking, which would be more attentive than CTM walking, showed broader brain activation as well as significant oxyHb increase.

There was an fNIRS study that observed increased PFC activity during CTM walking compared with overground walking in elderly [56], and this observation supports that overground walking would have less attentional resources than CTM walking. On the other hand, our study shows that ITM walking, which was to make it as similar as possible to overground walking in terms of physical activity, causes more attention than CTM walking in young healthy subjects. In spite of the different population of those studies, one can expect that the improved attention of ITM does not come from its physical function (self-paced treadmill) but from its training environment (protocol with appropriate biofeedback).

The proposed protocol of ITM-based locomotion training consists of acceleration, maintaining speed, and deceleration (Fig. 2b) [8, 9, 21]. Hence, we designed the walking phase of the block paradigm to contain those components (Fig. 3). Here, the maintaining speed period of the walking phase was much longer than acceleration or deceleration periods. This was done to mainly focus on investigating the effect of the ITM on the user’s attention in the maintaining speed period. Even though the rapid acceleration and deceleration are very stimulating periods, the maintaining speed period of the ITM protocol is usually much longer than the acceleration or deceleration periods.

In the experiment, we conducted ITM and CTM walking under two conditions which included slow and fast walking speeds. Since the conditions were adopted to investigate the effect of the task complexity felt by the user, those speeds for each subject were determined based on his or her preferred walking speed. The variability of the preferred speeds displayed in Fig. 4 shows that it is appropriate to consider each subject’s individual gait capacity, which is closely related to brain activation [43]. Using fixed speeds for all subjects could not achieve this.

From this study, it was verified that ITM-based locomotion training can result in more attentive gait training. This result shows that ITMs could be a promising solution to overcome the limitations of existing TBLT, especially regarding mental activity. This study still have some rooms to be improved. Although the expected target users of ITM-based locomotor training are elderly and patients with gait disorder, the hypothesis was validated with twenty young healthy subjects only. Moreover, due to the limitation of our fNIRS equipment, we did not use the short-separation channels, which is a promising method to correct the fNIRS signal [32, 57]. In the future, similar verification of ITMs with the short-separation channels should be performed with elderly and the patients, especially with central nervous system injuries to consider different activation patterns due to their brain lesions [25]. In addition, to investigate the gait recovery caused by the ITM-based locomotion training, the change of gait performance with ITM training needs to be evaluated by using an ambulatory gait monitoring system [58].

## Conclusion

By comparing the ITM walking with the CTM walking using fNIRS, we found that 1) the ITM causes significantly more attentive walking than the CTM and 2) the level of attention due to the ITM is affected by the target walking speed used. Along with kinematic/kinetic similarities, this attentive (non-automatic) training due to ITM-based locomotion training would result in better recovery of gait function for patients with central nervous system injuries.

## Abbreviations

CTM: conventional treadmill
Cz: cranial vertex
fMRI: functional magnetic resonance imaging
fNIRS: functional near-infrared spectroscopy
GLM: general linear model
Hb: hemoglobin
IRB: institutional review boards
ITM: interactive treadmill
oxyHb: oxygenated hemoglobin
PET: positron emission tomography
PFC: prefrontal cortex
PMC: premotor cortex
ROI: region of interest
SMA: supplementary area
SMC: sensorimotor cortex
TBLT: treadmill-based locomotion training
